# Comparative proteome analysis between ***C****. briggsae* embryos and larvae reveals a role of chromatin modification proteins in embryonic cell division

**DOI:** 10.1038/s41598-017-04533-8

**Published:** 2017-06-27

**Authors:** Xiaomeng An, Jiaofang Shao, Huoming Zhang, Xiaoliang Ren, Vincy Wing Sze Ho, Runsheng Li, Ming-Kin Wong, Zhongying Zhao

**Affiliations:** 1 Department of Biology, Hong Kong Baptist University, Hong Kong, China; 2 Biosciences Core Laboratory, King Abdullah University of Science and Technology, 23955-6900 Thuwal, Saudi Arabia; 3 Core Laboratory, The University of Hong Kong-Shenzhen Hospital, Shenzhen, China; 4 Department of Bioinformatics, School of Basic Medical Sciences, Nanjing Medical University, Nanjing, China

## Abstract

*Caenorhabditis briggsae* has emerged as a model for comparative biology against model organism *C. elegans*. Most of its cell fate specifications are completed during embryogenesis whereas its cell growth is achieved mainly in larval stages. The molecular mechanism underlying the drastic developmental changes is poorly understood. To gain insights into the molecular changes between the two stages, we compared the proteomes between the two stages using iTRAQ. We identified a total of 2,791 proteins in the *C. briggsae* embryos and larvae, 247 of which undergo up- or down-regulation between the two stages. The proteins that are upregulated in the larval stages are enriched in the Gene Ontology categories of energy production, protein translation, and cytoskeleton; whereas those upregulated in the embryonic stage are enriched in the categories of chromatin dynamics and posttranslational modification, suggesting a more active chromatin modification in the embryos than in the larva. Perturbation of a subset of chromatin modifiers followed by cell lineage analysis suggests their roles in controlling cell division pace. Taken together, we demonstrate a general molecular switch from chromatin modification to metabolism during the transition from *C. briggsae* embryonic to its larval stages using iTRAQ approach. The switch might be conserved across metazoans.

## Introduction

Regulation of protein expression is a fundamental biological process during metazoan development in which a single-cell zygote undergoes a series of divisions followed by cell fate differentiation, enabling formation of a functional organism. Expression dynamics of regulatory proteins plays a pivotal role in ensuring proper cell division and cell fate differentiation. Aberration in the dynamics of these proteins is often blamed for various developmental defects or an animal death. Over decades, many efforts have been made to investigate the temporal and spatial gene expression dynamics across species, including nematodes^1–3^. Regulatory proteins including transcription factors and the components of various signaling pathways are well-known for their roles in orchestrating organism development through transcriptional, translational or posttranslational control. Most studies on systematic gene expression focus on transcriptional dynamics^4, 5^. In addition, previous studies have shown that the mRNA level of a gene is often poorly correlated with its protein level spatially and temporally *in vivo* ^6, 7^, indicating a systematic analysis of protein dynamics is necessary for a better understanding of their function especially for those regulatory ones during animal development.

The nematode *C. elegans* is one of the well-established model organisms for studying genetic control of cell fate specification due to its short life span, simple and transparent body, and invariant development. It has been widely used in studying the molecular mechanisms of tissue formation and organogenesis^8–10^, sex determination^11^, programmed cell death^12^ and cell division asymmetry^13, 14^. *C. briggsae* is another nematode closely related to *C. elegans* ^15^, which provides an excellent model for comparative and evolutionary studies^16^. In addition to their similar developmental patterns, the morphology of *C. briggsae* is almost indistinguishable from that of *C. elegans*. For example, the cell division timings and cell fate specification are nearly identical^17–19^. Nevertheless, the two species are separated from their common ancestor 30–100 million years ago^20, 21^. Recently, empowered by various genetic and molecular tools^22^, *C. briggsae* has been developed as an attractive model for dissecting the genetic or molecular mechanisms of interspecific hybrid incompatibilities^23–25^, which is not possible using *C. elegans*. This is mainly due to a lack of a sister species with which *C. elegans* can mate and produce viable hybrid progeny; whereas such a species is identified for *C. briggsae* ^26^.

*C. briggsae* protein dynamics across developmental stages was previously characterized by Stable Isotope Labelling with Amino acids in Cell culture (SILAC)^27^. Previous studies using cell culture demonstrated that a newly developed proteomics approach using Isobaric Tags for Relative and Absolute Quantitation (iTRAQ) outperforms SILAC in protein identification^28^. In this study, we investigated protein dynamics between the embryonic and two larval stages using iTRAQ and contrasted the results obtained using the two approaches. As *C. elegans*, *C. briggsae* develops from an embryo into adulthood through four larval stages, i.e., L1, L2, L3 and L4 that are punctuated by each molting event. Its embryonic development involves multiple rounds of rapid of asynchronous cell division that is concomitant with cell fate differentiation. A previous study showed that the most drastic change in protein expression was observed between the embryo and L1 larval stage in *C. elegans* ^29, 30^. To characterize the changes in protein expression at systems-level between embryonic and larval stages of *C. briggsae*, we generated protein expression profiles for the mixed-staged embryos and larvae, including those from the first larval stage (L1) and the last larval stage (L4), using iTRAQ for relative protein quantization. Totally we identified 2,793 proteins in these three stages, 247 of which were found to undergo up- or downregulation between the embryo and two larval stages; while the expression level is much similar between L1 and L4 stage. Functional analysis of a subset of the upregulated embryonic proteins demonstrated a requirement of chromatin modification proteins for regulating cell division pace and temporal asymmetry during the *C. briggsae* embryogenesis.

## Results and Discussion

### Protein identification and characterization of DEPs (Differentially Expressed Proteins) between embryos and larvae

**Figure 1:**
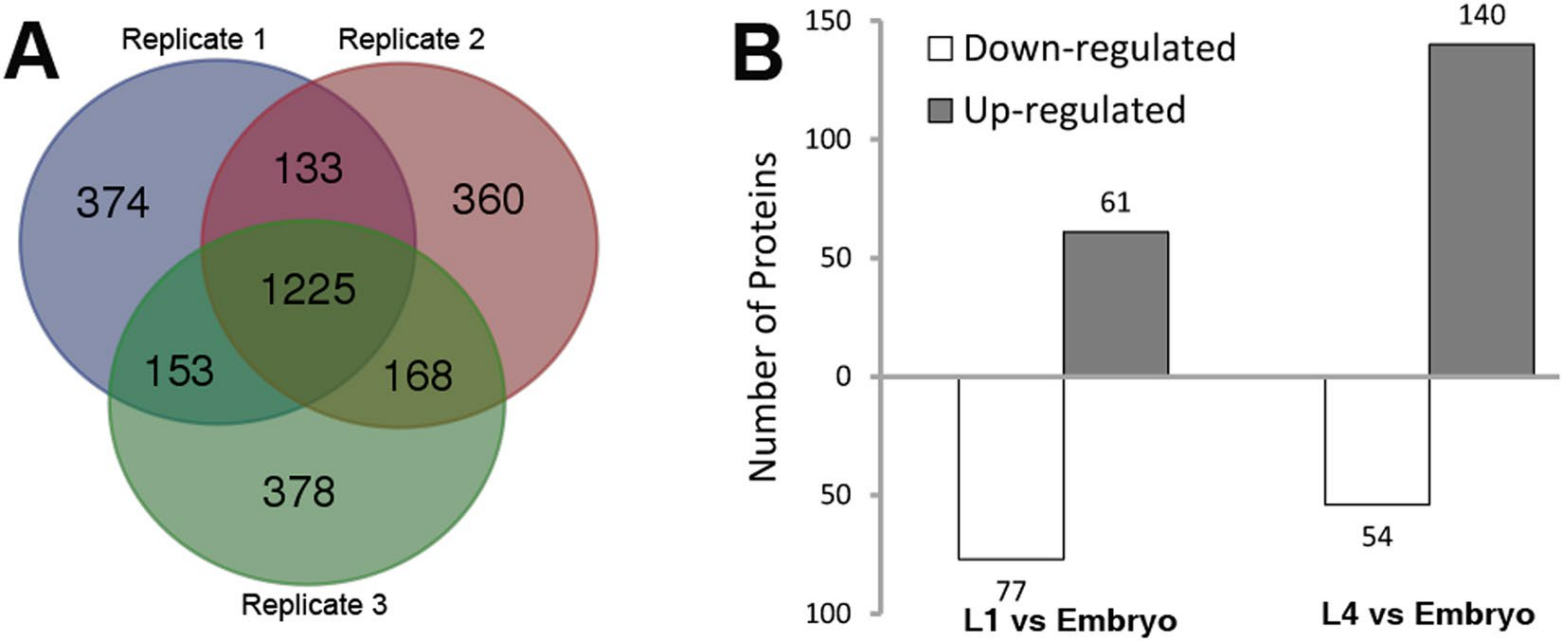
Comparison of proteomes identified by iTRAQ between three different replicates. (**A**) A Venn diagram showing the number of unique or shared proteins identified in three biological replicates. (**B**) Number of DEPs between embryonic and two larval stages (L1 and L4).

In this study, we tried to identify DEPs between embryonic and two larval stages in *C. briggsae* using iTRAQ-based quantitative proteomic approach, with a focus on identification of regulatory proteins involved in *C. briggsae* embryogenesis and larval development. Protein extracts from the *C. briggsae* mix-staged embryos, and two larval stages, L1 and L4, were prepared for iTRAQ analysis. Three biological replicates for each stage were subjected to multiplex iTRAQ assays. Only proteins identified at >95% confidence (peptide FDR <5%, p < 0.05 based on a decoy search) by at least two individual peptides were counted. There were a total of 1,885, 1,886 and 1,924 proteins that were identified in the replicate 1, 2 and 3, respectively (Fig. 1A). Of these proteins, 1,358 were shared between the replicate 1 and 2, 1,393 between the replicate 2 and 3, 1,378 between the replicate1 and 3, and 1,225 between all three replicates. A total of 2,791 proteins were identified in the combined dataset, 2,782 of which have quantitative information (Fig. 1A and Table S2).

The embryo sample was used as a reference, and the samples from the two larval stages, i.e., L1 and L4, were compared against it to identify the DEPs between the embryonic and larval stages. We defined an up- or a downregulated protein in the larvae versus the embryos using the similar threshold cutoff as those used previously^31–33^, i.e., when a protein abundance shows a ratio of 1.3-fold higher or 0.8-fold lower in the larvae than in the embryos in at least two replicates. A total of 247 proteins showed a differential expression between the embryonic and either of the two larval stages. Specifically, the expression of 61 and 77 proteins was up- or down-regulated in the L1 animals compared with the embryos, respectively; the expression of 140 and 54 proteins was up- or down-regulated in the L4 animals compared with the embryos, respectively (Fig. 1B). Surprisingly, 246 out of the 247 proteins exhibited the same trend in expression change between either L1 or L4 stage and embryo, with only a single protein showing an opposite dynamic trend between the two larval stages (Fig. 1B and Table S4), indicating a continuation of physiology between L1 and L4 stage but a drastic molecular change between the embryonic and the larval stages.

**Figure 2:**
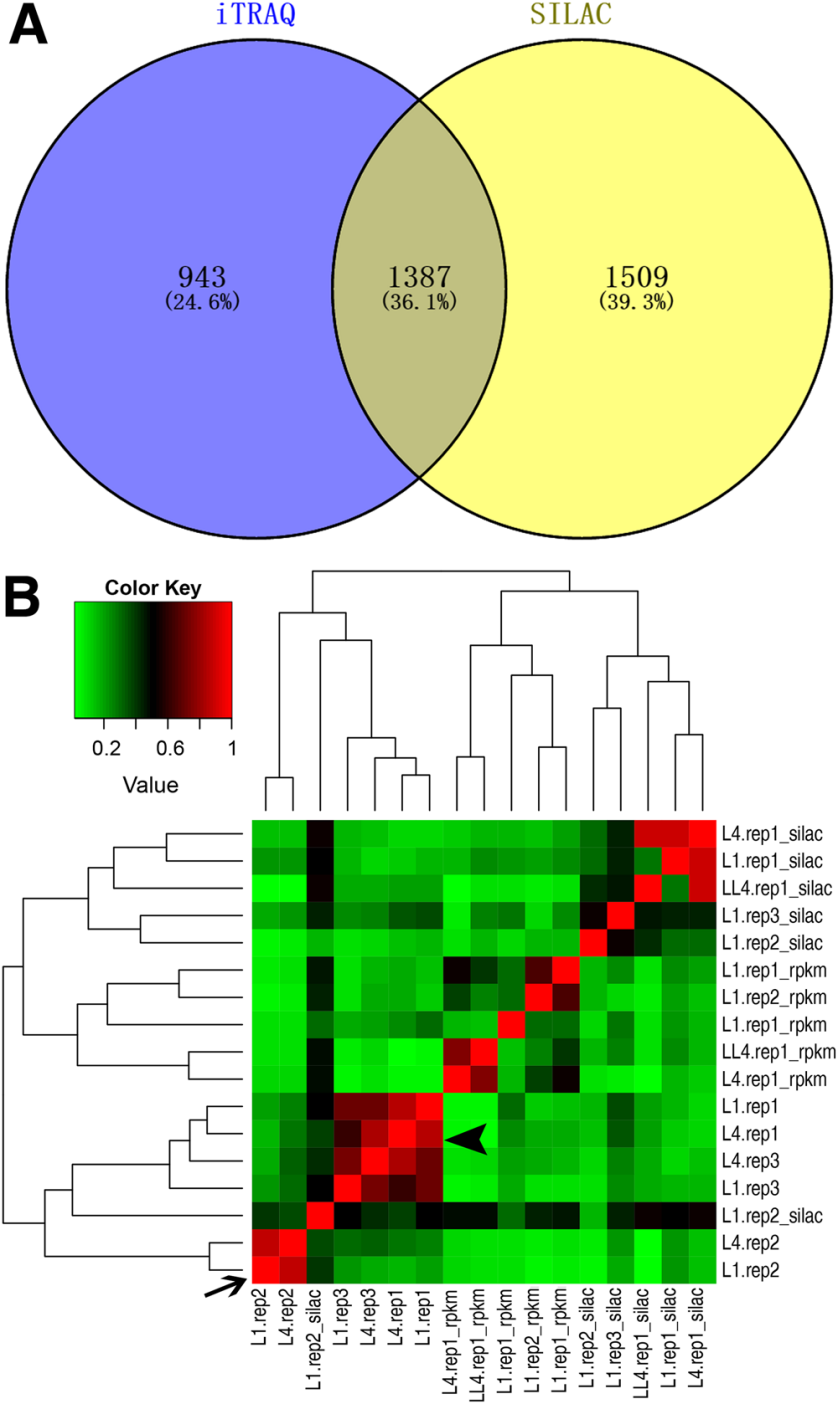
Comparison of *C. briggsae* proteomes identified by iTRAQ in this study and those by SILAC in a previous study^29^. (**A**) Venn diagram showing the total number of proteins identified by iTRAQ (blue) or SILAC (yellow) in all three replicates of L1 and L4 larval stages. Note only about a third of the proteomes is shared between the two methods. (**B**) A heat map showing the correlation of fold changes of the proteins/transcripts in larval stages versus embryonic stages identified by iTRAQ or SILAC. Protein or transcript samples measured in the previous study are indicated with “silac” or “rpkm”, respectively. Correlations of fold changes detected by iTRAQ between L1 and L4 stages are indicated with an arrowhead or an arrow for replicates 1 and 3 or replicate 2, respectively.

Given the different performances between iTRAQ and SILAC in protein identification^28^, we contrasted the proteins and their abundance changes in larvae versus embryos detected in this study using iTRAQ and those in a previous study using SILAC^29^. A total of 2330 and 2896 proteins were identified in L1 and L4 larvae by iTRAQ and SILAC, respectively (Fig. 2A, Table S2). However, only approximately a third of them are shared between the two, suggesting that the two methods are unique in their own ways in protein identification. To further compare the protein abundance changes detected by the two methods, we found that the proteins fold changes between larval and embryonic stages showed little correlation (Fig. 2B). Notably, overall fold changes of proteome in the two larval stages relative to the embryonic stage show high a correlation with a correlation coefficient over 0.7. Only modest correlation was observed between one replicate derived from SILAC (L1.rep2_silac) and our larval data (Fig. 2B). As reported previously^29^, the fold change in our proteome and that of the transcriptome showed little correlation. Given the differential results in both protein identification and its abundance between the two methods, it is necessary to further characterize the underlying mechanism that leads to the discrepancies, which is beyond the scope of this study. This study also demonstrated that a combined assay with both approaches substantially increased the number of identified proteins.

### GO and KOG analysis of differentially expressed proteins

**Figure 3:**
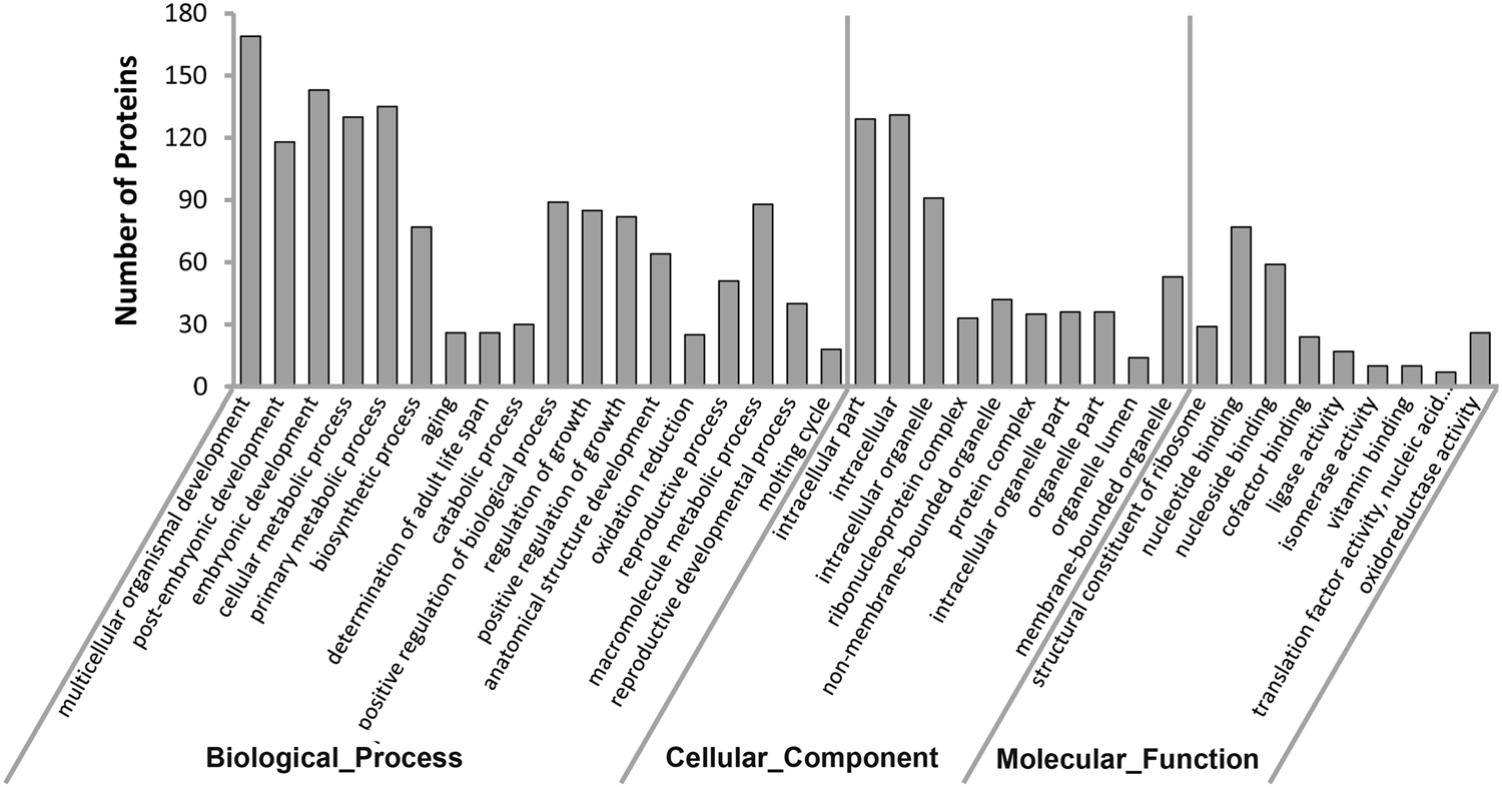
A histogram showing the enriched GO terms (Biological_Process, Cellular_Component, and Molecular_Function) for the DEPs identified with iTRAQ.

To obtain a general idea of how the identified 246 DEPs that are shared between L1 and L4 stage function during transition from *C. briggsae* embryonic to larval stages, we carried out GO analysis using DAVID^34^. Our results showed that 18, 10 and 9 GO categories were significantly enriched in terms of “biological-process”, “cellular-component”, and “molecular function”, respectively (Fig. 3). For those proteins significantly enriched in “biological process”, most of them were involved in the “multicellular organismal development” (169, 68.4%), or “embryonic development” (143, 57.9%), which is consistent with our experimental design, i.e., capturing the differentially expressed proteins between embryonic and larval stages. For those proteins significantly enriched in “cellular component”, proteins with the most pronounced enrichment are those of “intracellular part” (129, 52.3%), suggesting that secretory proteins, for example, signaling proteins, are poorly enriched. For those proteins significantly enriched in “molecular function”, proteins with the most pronounced enrichment are those related to “nucleotide binding” (77, 31.2%) and “nucleoside binding” (59, 23.9%) (Table S5), demonstrating that regulatory proteins such as chromatin modifiers or transcription factors are more likely to be differentially expressed between embryonic and larval stages. An enrichment in regulatory DNA binding proteins in DEPs is expected. This is because during *C. briggsae* embryogenesis, cells undergo fast division and most of the cells are differentiated into their final fates, a process heavily depending on the regulatory factors, including DNA-binding ones such as chromatin modifiers and transcription factors.

**Figure 4:**
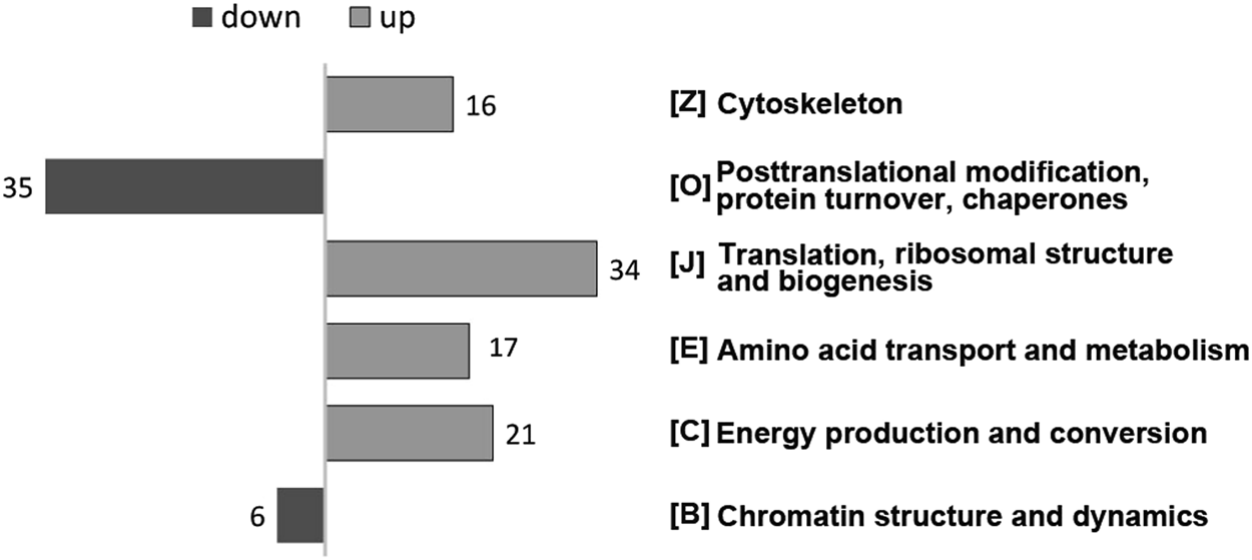
A histogram showing the enriched KOG function classes of the identified proteins as in Fig. 3.

To further explore the functional insight of the DEPs during *C. briggsae* development, all the DEPs were searched against the EuKaryotic of Orthologous Group (KOG) database. The KOG analysis was preferred as it allows a simple and straightforward search for a conserved core of a large set of essential eukaryotic genes that has arisen in the evolution of the eukaryotic genome^35^. As a result, 225 out of the 246 DEPs can be grouped into 23 KOG categories (Table S5). The top three enriched categories were “[O] for posttranslational modification, protein turnover, chaperones”, “[J] for translation, ribosomal structure and biogenesis”, and “[C] for energy production and conversion” (Fig. 4). However, there is a drastic difference between up- and down- regulation in the top six KOG categories based on the protein count (Table S5). Four of them were significantly up-regulated in the two larval stages compared with the embryonic stage, including (i) [C] energy production and conversion, (ii) [E] amino acid transport and metabolism, (iii) [J] translation, ribosomal structure and biogenesis, and (iv) [Z] cytoskeleton. The remaining two classes were down-regulated in the two larval stages compared with the embryonic stage, including (i) [B] chromatin structure and dynamics, and (ii) [O] posttranslational modification, protein turnover, chaperones (Fig. 4), which is consistent with the enrichment analysis for “molecular function” with DAVID (Fig. 3). The results indicate that *C. briggsae* animals invest heavily in proteome on cell growth during larval development while their proteome undergoes more frequent regulatory changes in an embryo than in postembryonic development.

Our results demonstrated a sharp difference in protein abundance between *C. briggsae* embryonic and larval stages, whereas the protein abundances are mostly comparable between the two larval stages though their correlation with transcript level is not obvious (Fig. 2B). The proteins required for energy production, amino acid metabolism, protein translation, and cytoskeleton assembly are expressed at a higher level in the larvae than in the embryos, suggesting a higher demand for energy and biogenesis to sustain larval development^36^. In contrast, the proteins required for chromatin dynamics, gene regulation and post-transcriptional modification are expressed at a higher level in embryo than in the larval stages. Regulatory proteins, including histone modifiers, transcription factors, and chromatin remodeling factors, play a major role in defining chromatin dynamics and therefore gene expression level. Presumably, most of the regulatory changes in development take place during embryogenesis, including asymmetries in cell fate and cell division timing, cell migration and morphogenesis, transition from maternal to zygotic expression, all of which demand seamless coordination. Coordination between these processes is critical for ensuring proper development in particular during metazoan embryogenesis^13^. Our comparison of proteomes between the embryo and larva indicates a differential need in protein expression to accommodate the transition from embryogenesis to larval development.

**Figure 5:**
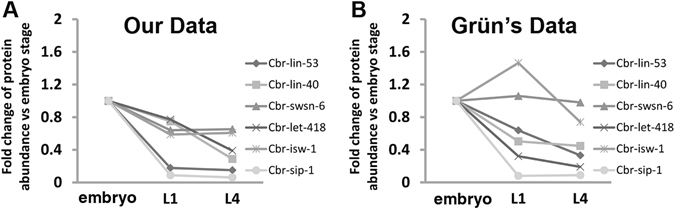
Expression dynamics of a subset of six chosen proteins that are up-regulated in embryo across different developmental stages, including embryo, L1, and L4 stage. (**A**) Data from this study. (**B**) Data from a previous study^29^.

As mentioned above, our proteomics data using an iTRAQ analysis do show deviations from those using a SILAC assay (Fig. 2B)^29^. For example, we showed that all six chromatin modifiers identified by Grün *et al*. were expressed at a lower level in the larval stages than in the embryonic stage (Fig. 5A); whereas the Grün’s data demonstrated a higher expression of *cbr-let-418* and *cbr-swsn-6* in L1 larva than in embryo (Fig. 5B). We speculate that the discrepancy is likely produced by the technical differences between the two approaches, highlighting a need for future characterization of the reproducibility in protein identification and quantification between the technical replicates from iTRAQ and SILAC.

It is worthy of note that despite thousands of proteins were identified in this study, most of these proteins are relevant to development or physiology and are likely to be conserved across species. Many other proteins that are only conditionally expressed would be missed out in our analysis. For example, a subset of 108 proteins was differentially expressed in *C. elegans* upon exposure to pathogen *Staphylococcus aureus* using 2D gel analysis followed by mass spectrometry. Only 9% of the identified protein was also found in *C. elegans* infected with another pathogen, *Aeromonas hydrophila* ^37^. Similar analyses in *C. elegans* infected with pathogens *Proteus mirabilis* or *Vibrio alginolyticus* demonstrated differential sets of proteins in the immunity against the respective pathogens^38, 39^. Given most of the genes involved in pathogen infection or host immunity are fast evolving^40^, they are specifically expressed upon exposure to unique pathogens, and very likely to be missed out in our assay in laboratory cultures.

### Functional relevance of DEPs in embryogenesis

Given a higher level of a subset of proteins in the embryos than in the larvae which are known to be involved in chromatin dynamics (Figs 3 and 4, Table S5), we set to investigate their global roles in regulating asymmetry of division timing during the proliferative stage of *C. briggsae* embryogenesis. This was achieved using RNAi followed by automated cell lineaging analysis^17^ which allows for systematic measurement of cell division timings and identification of cell identities. A total of seven proteins that showed a higher level of expression in the embryos were chosen for the lineage analysis, including five proteins required for chromatin structure and dynamics (*cbr*-*lin-53*, *cbr*-*lin-40*, *cbr-let-418, cbr-swsn-6*, *cbr-isw-1*) and two proteins necessary for posttranslational modification (*cbr-sip-1*, *cbr-smo-1*). We chose these genes because depletion of their orthologues in *C. elegans* all produced defects in division asynchrony during embryogenesis^13^.

**Figure 6:**
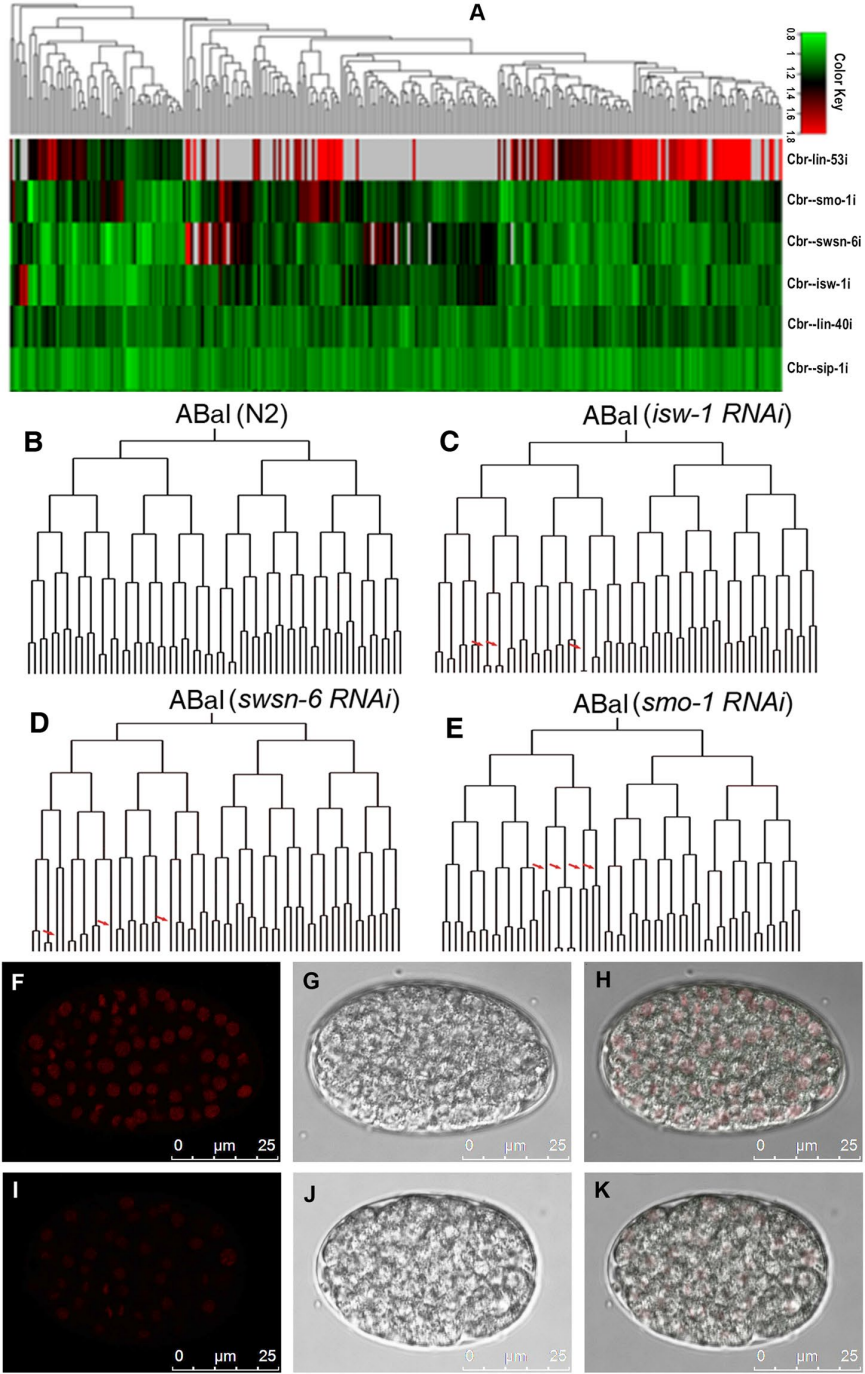
Roles of seven embryonically up-regulated proteins in cell division timing during *C. briggsae* embryogenesis. (**A**) Hierarchical clustering of cell division timings of RNAi embryos with genotypes indicated on the right. Up-regulation in division paces is colored in red and black and down-regulation in division paces in green. Cells absent in an RNAi embryo were shown in grey. (**B**–**E**) Lineage trees of a wildtype and RNAi embryos rooted with cell “ABal” up to roughly 350-cell stage with horizontal line indicating a cell division and vertical line division timing. For a full lineage tree rooted with a zygote, see Fig. S1. Significant changes in cell division timing relative to wildtype (N2) are indicated by a red arrow. The genotypes are shown above the tree. (**F**–**K**) Epifluorescence, Nomarski and their superimposed micrographs for a wildtype (**F**–**H**), approximately 350-cell stage) and a *let-418* RNAi embryo (arrested at approximately 200-cell stage), respectively. Note the reduced expression intensity of histone marker (HIS-72::mCherry) before (**F**) and after (**I**) the RNAi.

RNAi against *cbr-lin-53*, *cbr-let-418, cbr-swsn-6*, *cbr-isw-1*, and *cbr-smo-1* produced high penetrance of embryonic lethality while RNAi against the remaining two produces superficially wild type phenotypes (Table 1). To systematically characterize the detailed defects at cellular level throughout embryogenesis, we performed RNAi against all these genes by microinjection followed by 3D time-lapse imaging. We collected confocal time-lapse fluorescence micrographs of a developing embryo expressing a P*cbr*-*his-72*:HIS-72::mCherry fusion protein as a lineaging marker^17^. Although we collected the images over 550-cell stage, we chose to routinely curate all the images up to approximately 350 cells due to crowdedness of nuclei and a poor image quality beyond this stage^41^. We quantified division timings for each cell for both wildtype and perturbed embryos in three replicates using software as described^17, 42^. Division timings for a total of 12 wildtype embryos were collected and used as a control. The results demonstrated that RNAi against all seven genes led to severe defects in asymmetries of cell division timing or in overall division pace except those from RNAi against *cbr-lin-40*, and *cbr-sip-1* (Table 1, Fig. 6, Fig. S1).

**Table 1:** Summary of RNAi phenotypes for the selected genes in lineage analysis.

Class	Gene name	Gene function	Overall phenotype	Lineage phenotype
Chromatin structure and dynamics	*cbr-lin-53*	Nucleosome remodeling factor	Embryonic lethality	Slower division
	*cbr-swsn-6*	SWI/SNF nucleosome remodeling complex component	Embryonic lethality	Division symmetry breaking
	*cbr-let-418*	Predicted helicase	Embryonic lethality	Lineaging marker depletion
	*cbr-lin-40*	Histone deacetylase complex	Wildtype	Wildtype
	*cbr-isw-1*	Chromatin remodeling complex NURF	Wildtype	Division symmetry breaking
Posttranslational modification	*cbr-smo-1*	Ubiquitin-like proteins	Embryonic lethality	Division symmetry breaking
	*cbr-sip-1*	Heat shock protein	Wildtype	Wildtype

RNAi against *Cbr-lin-53* caused an overall reduction in division paces, early embryonic arrest and missing of many cells at the developmental stage comparable to that of the wild type. A previous study showed that inhibition of its homolog *lin-53* in *C. elegans* exhibited severe defects in chromosome segregation^43^, which could be responsible for the observed defect in cell division pace. RNAi against *cbr-isw-1*, *cbr-swsn-6*, and *cbr-smo-1* showed defective asymmetries in cell division timing for a subset of cells, including cell-specific slowdown in division pace, establishment of a novel division asymmetry or breaking of existing division symmetry (Table 1, Fig. 6A–E, Fig. S1). *Cbr-swsn-6* encodes an orthologue of putative component of SWI/SNF nucleosome remodeling complex. Inhibition of its expression abolished division asymmetry or delayed division in many ABa progeny around 350-cell stage (Fig. 6D, Fig. S1), suggesting that *cbr-swsn-6* is selectively required for normal cell cycle progression in these cells. *Cbr-isw-1* encodes a chromatin remodeling protein within a Nucleosome Remodeling Factor (NURF)-like complex. RNAi against *cbr-swsn-6* produced defects in cell division pace similar to those of RNAi against *cbr-swsn-6* although mostly in different cells (Fig. 6C), suggesting a synergetic role between *cbr-swsn-6* and *cbr-isw-1* in setting cell-specific division paces and asymmetries. *Cbr-smo-1* encodes the orthologue of SUMO1, a small ubiquitin-like moiety that is an important posttranslational modifier involved in the regulation of various cellular processes^44^. Its *C. elegans* equivalent, *smo-1* has been demonstrated to be required for normal embryogenesis in *C. elegans* ^45^. Our study supports the essential role of *cbr-smo-1* in embryogenesis in *C. briggsae* as judged by the fact that *cbr-smo-1* RNAi produced high penetrance of embryonic lethality. Further study using automated lineaging technique showed the lethality is likely due to the delay of cell division pace in a subset of cells in ABala lineage (Fig. 6E). *Cbr-let-418* is predicted to encode a subunit of nucleosome remodeling and histone deacetylase (NURD) complex. RNAi against this gene resulted in a global depletion of expression of the lineaging marker, cbr-HIS-72::mCherry and embryonic arrest at approximately 200-cell stage (Fig. 6F to K), which prevented it from automated lineaging analysis, suggesting its critical role in regulating overall gene expression. Only modest phenotypes in cell division timing were observed in the RNAi embryos against *cbr-lin-40* and *cbr-sip-1* (data not shown), suggesting they might be functionally redundant with its paralogs.

In summary, we identified a total of 247 DEPs between *C. briggsae* embryonic and two larval stages by iTRAQ analysis. KOG analysis showed that the proteins relevant to chromatin dynamics, posttranslational modification and turnover are preferentially expressed in the embryonic stage than in the larval stages; whereas those relevant to energy production, amino acid transport and cytoskeleton are enriched in larval stages. The results support a more active chromatin modification and dynamics in embryogenesis than in postembryonic development, which likely serves to coordinate cell division pace and cell fate specification. A higher demand for energy production and metabolic rate in larvae is consistent with the fact that cell growth is major task during larval development, whereas most of cell division and cell fate specification are completed during embryogenesis. In summary, we demonstrated an overall molecular switch from chromatin modification to metabolism during the transition from *C. briggsae* embryonic to its larval stages using iTRAQ approach. The function and expression dynamics of the identified proteins are likely applicable to many other metazoan species.

## Methods

### Worm maintenance and sample preparation

Wild type *C. briggsae* (AF16) animals were used for all protein extractions. A GFP-expressing *C. briggsae* transgenic strain, RW20045 (*cbr-unc-119*, *stIs20025*[cbr-HIS-72::mCherry], *stIs20045*[PHA-4::GFP, *unc-119* (+)]), was constructed previously^17^ and was used for cell lineage analysis as detailed below. *C. briggsae* strains were cultured on NGM plate seeded with OP50 *E. coli* at room temperature unless specified otherwise^46^. About 1 ml of gravid adult worms were treated with alkaline bleach to isolate embryos as described^47^. Half of the embryos were used for protein extraction and proteomics analysis, while the remaining half embryos were incubated in M9 buffer at room temperature overnight to allow for hatching and arrest at L1 stage. The starved, synchronized L1 larvae were transferred onto NGM plates seeded with OP50 and cultivated at 24 °C. L1 and L4-staged larvae were collected 4 and 48 hours after incubation, respectively. The worms were washed three times in M9 buffer, and pelleted by centrifugation at 3,000 rpm for 2 min. 1 mL of lysis buffer (50 mM HEPES, 1 mM EGTA, 1 mM MgCl2, 400 mM KCl, 10% glycerol, pH7.4, with protease inhibitors) was added for every 100 μL worm pellet. Samples were sonicated on ice, and centrifuged at 13,000 rpm for 20 min. Three biological replicate samples were prepared for each stage.

### Protein extraction, digestion, and iTRAQ labeling

The supernatants were collected and their protein concentrations were determined using RC DC™ Protein Assay kit (Bio-rad, Berkeley, CA, USA). Approximately 100 μg proteins of each sample were reduced with 5 mM tris-carboxyethyl phosphine hydrochloride for 60 min at 37 °C, alkylated with 10 mM methylethanethiosulfonate for 20 min at room temperature (RT). The reaction mixtures were diluted ten times with deionized water followed by digestion with trypsin (Promega, Madison, WI, USA) overnight at 37 °C at a 1:50 trypsin-to-protein mass ratio. The digests were dried using a SpeedVac (Eppendorf, Hamburg, Germany) and labeled with 4plex iTRAQ reagents (Applied Biosystems, Framingham, MA, USA) according to the manufacturer’s protocol. The 4plex iTRAQ reagents contain four different stable-isotope covalent mass tags 114, 115, 116, and 117. The samples from embryos, L1 and L4 worms were labeled with the mass tags 114, 115 and 116, respectively. Peptide samples were reconstituted in 30 μL of 0.5 M TEAB buffer and mixed with 70 μL of ethanol suspended iTRAQ reagents (one iTRAQ reporter mass tag per peptide sample). Labeling reactions were carried out at RT for 60 min before all four samples were mixed into a single tube and dried using a SpeedVac.

### Peptide fractionation and MS analysis

The combined iTRAQ-labeled samples were fractionated using strong cation exchange chromatography as described^48^). Each fraction was analyzed twice on LTQ-Orbitrap Velos (Thermo Scientific, Germany) coupled with an Easy-nLC (Thermo Scientific). The sample was injected and concentrated in a preconditioned column (0.3 × 50 mm) packed with C18 AQ (5 μm particles, 200 Å pore size)(Bruker-Michrom, Auburn, CA, USA). The peptide separation was performed in a preconditioned capillary column (0.1 × 150 mm, with C18 AQ of 3 μm particles and 200 Å pore size)(Bruker-Michrom). The peptide was separated using a 60-min gradient comprised of 35 min of 0–35% mobile phase B (0.1% formic acid in ACN), 10 min of 35–80% B, and 15 min of 80% B. The total flow rate of the gradient was set at 40 nL/min. The sample was introduced into LTQ-Orbitrap through a Nanospray Flex (Thermo Scientific) with an electrospray potential of 1.5 kV. The ion transfer tube temperature was set at 160 °C. The LTQ-Orbitrap was set to perform data acquisition in the positive ion mode. A full MS scan (350–1600 m/z range) was acquired in the Orbitrap at a resolution of 30,000 (at 400 m/z) in a profile mode, a maximum ion accumulation time of 1 second and a target value of 1 × e^6^. Charge state screening for precursor ion was activated. The six most intense ions above a 1000-count threshold and carrying multiple charges were selected for a paralleled fragmentation (MS/MS) by collision-induced dissociation (CID) in the linear ion trap and the higher energy collision dissociation (HCD) in the Orbitrap. Dynamic exclusion for both CID and HCD fragmentation was activated with a repeated count of 2, repeat duration of 30 s, exclusion duration of 45 s, and ±5 ppm mass tolerance. The additional CID settings included a maximum ion accumulation time of 200 milliseconds for MS/MS, a target value of 1 × e^4^, normalized collision energy at 35%, an activation Q at 0.25, isolation width of 3.0 and activation time of 10 millisecond. For HCD, the settings included a full scan with Orbitrap at a resolution of 7,500 (at 400 *mlz*) in a centroid mode, a maximum ion accumulation time of 200 millisecond for MS/MS, a target value of 5 × e^4^, a normalized collision energy at 40%, isolation width of 3.0 and activation time of 0.1 millisecond. Original proteomics data were submitted to ProteomeXchange via the PRIDE database with identifier PXD005549.

### Mass spectrometry data analysis

The data from three biological replicates were searched against SwissProt Database version 2.0 (*C. briggsae*: 21, 973 entries)^49^ and combined using Protein Pilot software 3.0 (Applied Biosystems) with background correction and bias correction turned off. Only peptides of confidence >50% were used for protein quantification, and a 95% confidence (unused prot score >1.3) threshold for protein identification as determined by Protein Pilot 3.0 was used for inclusion into the final protein report.

### Functional annotation and classification

*C. elegans* orthologs for identified *C. briggsae* proteins were used for all the Gene Ontology (GO) analysis due to the lack of an *C. briggsae* annotation for the DAVID database^34^. NIH DAVID Bioinformatics Database was used to determine the categories that were enriched for a given protein set. The 247 proteins that showed a change in abundance in larvae than in embryo were queried against the total identified proteins. Only a category with the Benjamin and Hochberg’s corrected *p*-value < 0.05 was considered as significantly enriched.

To categorize the proteins that were upregulated in the embryos, EuKaryotic of Orthologous Groups (KOG) annotation was carried out against the NCBI KOG database. The KOG annotations of differentially expressed proteins (DEPs) were classified into 22 groups, and the enrichment analysis was conducted through hypergeometric distribution testing using the “phyper” function in the R software package. The Bonferroni correction was used to adjust the *p*-values. The significantly enriched functional clusters were selected based on a corrected *p*-value (<0.05).

### dsRNA design and microinjection

Double-stranded RNA (dsRNA) primers were designed as described^13, 45, 50^. Briefly, primer pairs were selected to amplify 400–800 bp fragments, with preference given to the common regions of all isoforms. Primers used for dsRNA production were listed in Table S1. PCR reactions were performed using 1 μM primers and ~0.1 μg AF16 genomic DNA. 1 μl PCR product was used for transcription reaction using T7 RNA polymerase (NEB). The reactions were diluted to 300 ng/μl with TE buffer. dsRNAs were injected bilaterally into the gonads of young adult hermaphrodites, which were left at 24 °C for 24 hrs. Percentage of embryonic lethality was recorded. Embryos were then retrieved for 3D time-lapse imaging as described^51^.

### Progeny lethality test

Progeny lethality test was carried out as previously reported^45, 50^. In brief, ten injected worms were transferred to a new plate 24 hours after injection of the dsRNA and killed 8 hrs later. The plate was checked for the embryo count immediately and F1 development two days later. A dsRNA injection that gave rise to 0–10 eggs was classified as sterile. A dsRNA injection that gave rise to many eggs but only 0–10 F1 animals was classified as embryonic lethal.

### Imaging and automated lineaging

One to four-celled embryos were retrieved from the adults that had been subjected to injection for 24 hours. Embryos were mounted and imaged as described^52^. Imaging was performed with an inverted Leica SP5 confocal microscope, and image stacks were consecutively collected from a mCherry channel every 1.5 minutes with an “xy” frame size of 712X512 pixels and scanning speed of 800 Hz. mCherry was used as a lineaging marker. Fluorescence images from 41 focal planes were collected consecutively for three embryos per imaging session with a Z resolution of 0.71 μm from top to the bottom of an embryo for every time point. Images were collected for a total of 240 time points during which the cell count would reach approximately 550 in a wild-type embryo. The entire imaging duration was divided into four time blocks by time point, i.e., 1–50, 51–130, 131–200 and 201–240. Z axis compensation was 20–95% for the laser of 594 nm. The pinhole sizes for the four blocks were 1.6, 1.4, 1.0 and 0.8 AU (area unit), respectively.

Automated lineaging and gene expression profiling were performed as described^52, 53^. Specifically, raw fluorescence TIFF images acquired for lineaging marker were used as an input for lineaging algorithms^54, 55^ implemented on Linux operating system to automatically reconstruct cell lineage. The output of the automated lineaging was manually curated to correct StarryNite errors in a graphical interface, AceTree^56^. At least two embryos were curated for each gene up to approximately 350-cell stage or up to the last time point that an embryo was editable in a perturbed embryo.

### Statistical analysis

*C. briggsae* RNA-seq RPKM data and SILAC data are fetched from GSE53359^29^. The one-way hierarchical clustering was performed in R (version 3.0.1) with spearman’s rank correlation test. The heatmap showing the correlation was generated by heatmap.2 in R (version 3.0.1).

## Electronic supplementary material

Supplementary Table 1 and Figure 1

Supplementary Table 2

Supplementary Table 3

Supplementary Table 4

Supplementary Table 5
