# Supplementary material for: Comparative proteome analysis between C. briggsae embryos and larvae reveals a role of chromatin modification proteins in embryonic cell division
Source: Sci Rep. 2017 Jun 27;7:4296. doi: 10.1038/s41598-017-04533-8 (PMC5487359; doi:10.1038/s41598-017-04533-8)
Supplement: Supplementary file 1 — Supplementary Table 1 and Figure 1 [file 41598_2017_4533_MOESM1_ESM.pdf]

Comparative proteome analysis between *C. briggsae* embryos and larvae reveals a role of chromatin modification proteins in embryonic cell division

Xiaomeng An[†#1], Jiaofang Shao[†#2], Huoming Zhang[‡], Xiaoliang Ren[†], Vincy Wing Sze Ho[†], Runsheng Li[†], Ming-Kin Wong[†], Zhongying Zhao[†]*

[†]Department of Biology, Hong Kong Baptist University, Hong Kong, China; [‡]Biosciences Core Laboratory, King Abdullah University of Science and Technology, 23955-6900 Thuwal, Saudi Arabia.

[1]Current address: Core Laboratory, The University of Hong Kong-Shenzhen Hospital, Shenzhen, People's Republic of China; [2]Current address: Department of Bioinformatics, School of Basic Medical University, Nanjing Medical University, Nanjing, People's Republic of China

Table S1. List of primer sequences used for PCR amplification of dsRNA template.

	Forward Sequence	Reverse Sequence
cbr-lin-53	TAATACGACTCACTATAGGGacgaccagactgtatgtcactggga	TAATACGACTCACTATAGGGttcgtcgcgatgagattcgaacgaa
cbr-swsn-6	TAATACGACTCACTATAGGGttccagaagtcactccgagctacga	TAATACGACTCACTATAGGGgcaccgtattttcgttcggcttgag
cbr-let-418	TAATACGACTCACTATAGGGtaatggcgacccgttgaactctgac	TAATACGACTCACTATAGGGcggctgcagtttcggtattctcaga
cbr-lin-40	TAATACGACTCACTATAGGGcgaagatgcattggacaagtgtggc	TAATACGACTCACTATAGGGcagaatttcctgctgcagcctgttg
cbr-isw-1	TAATACGACTCACTATAGGGcgtgctggaggtcttggaatcaact	TAATACGACTCACTATAGGGtttccggctctcattgcttccttgt
cbr-smo-1	TAATACGACTCACTATAGGGagtaacgaggtccacttccgagtca	TAATACGACTCACTATAGGGgtaggggtgacgacggtaaaacagg
cbr-sip-1	TAATACGACTCACTATAGGGtgtccctcgatggtgagaatgttgc	TAATACGACTCACTATAGGGcgcggtcttccagaattgtctcgat

Table S2. List of identified proteins in all replicates by iTRAQ assay.

Table S3. List of the *C. briggsae* proteins specifically identified by iTRAQ compared to those by SILAC.

Table S4. List of identified DEPs in all samples.

Table S5. List of GO and KOG categories for the 247 differentially expressed proteins (DEPs) identified by iTRAQ between embryo and two larval stages (L1 and L4).

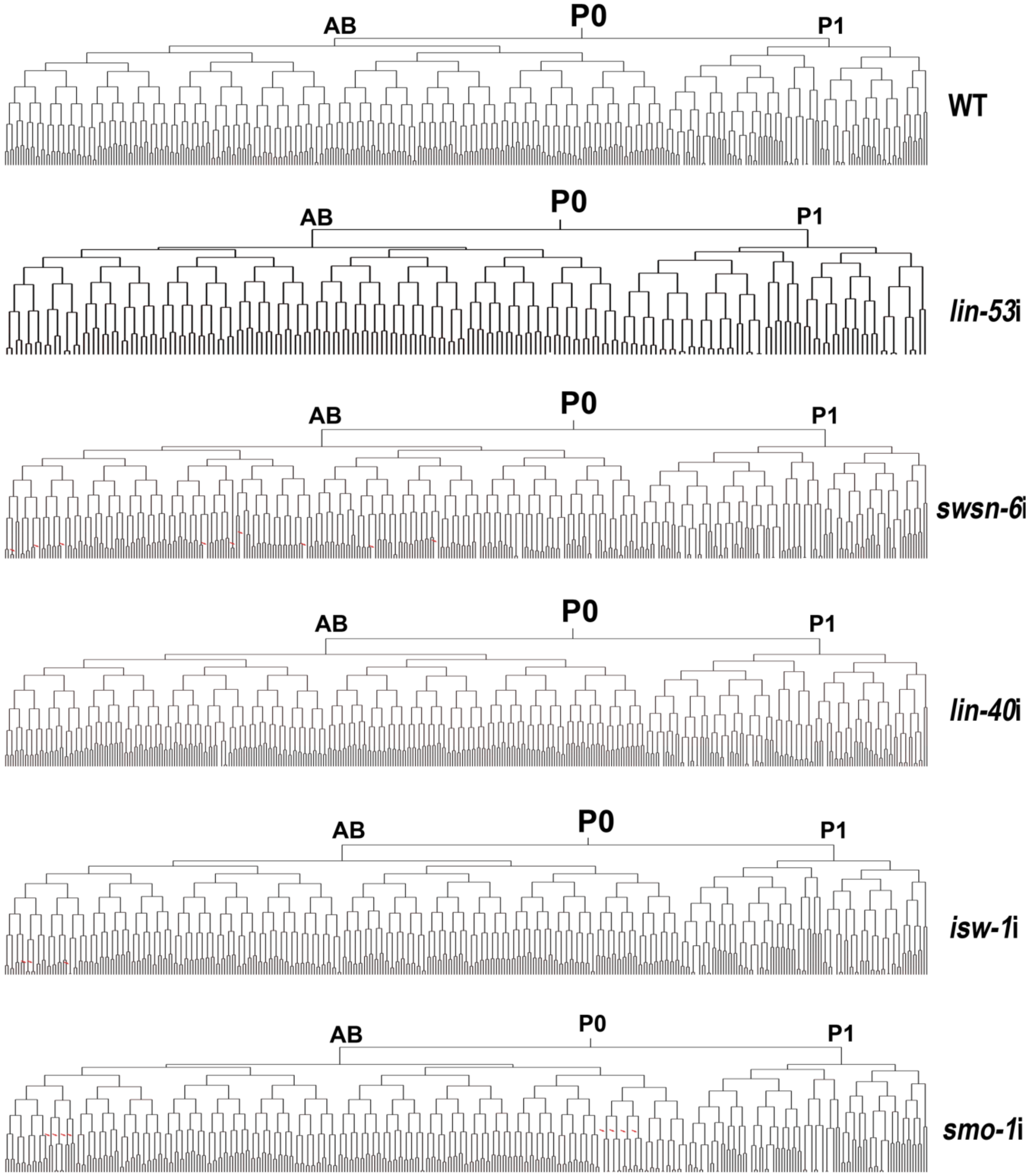

Fig. S1. Full cell lineage trees for embryos of wildtype and RNAi against five embryonically up-regulated genes. See also Fig 6 B-E.
